# Bridging physics and biology in acupuncture: flexible multimodal bioelectronics for decoding the deqi microenvironment

**DOI:** 10.3389/fbioe.2026.1881508

**Published:** 2026-07-24

**Authors:** Zhanhao Wu, Yinhu Hu, Lei Song, Juan Jin, Chan Zhu, Yalan Chen, Yiyang Zhang, Jie Zhuang, Gang Xu

**Affiliations:** 1 Department of Rehabilitation Medicine, The Second Rehabilitation Hospital of Shanghai, Shanghai, China; 2 College of Artificial Intelligence for Traditional Chinese Medicine, Shanghai University of Traditional Chinese Medicine, Shanghai, China

**Keywords:** acupuncture digital twins, deqi microenvironment, flexible bioelectronics, mechanotransduction, needling kinematics

## Abstract

Clinical standardization of acupuncture remains limited by its reliance on empirical tactile feedback and subjective patient sensations, a physiological response collectively termed Deqi. Here, the Deqi microenvironment refers to the local, time-varying tissue domain around the needle in which mechanical deformation, microvascular perfusion, biochemical mediator release and electrophysiological activity are coupled and measurable. Conventional rigid sensing architectures cannot decode this process without disturbing it, because their stiffness can create mechanical mismatch with soft tissues, distort dynamic signals and provoke non-physiological inflammatory artifacts at the biological interface. This review examines the evolution from conventional rigid instrumentation toward flexible, multimodal bioelectronics for high-fidelity quantification of needling kinematics. Sub-micron electrospun nanomeshes, liquid metals and low-dimensional carbon networks enable the decoding of manipulation dynamics without tactile interference. These conformal arrays also support real-time, *in situ* mapping of the Deqi microenvironment across tissue biomechanics, local hemodynamics, biochemical metabolism and electrophysiological signaling. The translation of these physical inputs into systemic physiological responses is mediated by structurally defined mechanotransduction pathways, including the peripheral force-immune axis regulated by Ptgs2+ telocytes and central neuroanatomical projections such as the PROKR2-dependent vagal-adrenal circuit. Synthesizing the resulting high-dimensional data streams requires specialized computational architectures. Graph neural networks, multimodal Transformers and related AI frameworks can synchronize heterogeneous sensor inputs and support the construction of a computable Deqi Index for cautious clinical prognostication. Future progress will depend on scalable manufacturing, chronic bio-interface stability and clinically governed closed-loop control. Taken together, the integration of intelligent robotics and Acupuncture Digital Twins may help transition acupuncture from a heuristic manual practice toward a quantifiable, data-driven medical intervention.

## 1 Introduction

Clinical acupuncture yields reproducible outcomes across diverse systemic pathologies. Its mechanical foundation, however, remains difficult to quantify precisely. The procedure operates through a coupled sensory loop. The practitioner adjusts manual force according to localized tissue resistance, while the patient reports a corresponding psychophysical reaction clinically defined as “Deqi” (Zhu et al., 2023). This dependence on heuristic perception sustains a persistent analytical black box. Standard clinical protocols lack the bio-instrumentation needed to record active needling kinematics and *in vivo* physiological tissue responses at the same time. Without these variables, clinicians cannot precisely calibrate a mechanical “intervention dose.” This measurement gap separates acupuncture from biomedical frameworks that require reproducible dose-response metrics. Transitioning the modality into computable digital medicine therefore requires high-spatiotemporal-resolution sensor architectures that translate subjective somatic thresholds into continuous physical parameters.

Addressing the discrepancy between empirical observation and biological quantification, this review conceptually redefines Deqi as a measurable, integrated biological milieu rather than a purely subjective psychological projection. Synthesizing current clinical and physiological evidence, we propose that the Deqi microenvironment is governed by four coupled biological dimensions. First, tissue mechanics is defined by extracellular matrix (ECM) remodeling and the needle-rotation-induced winding effect (Langevin et al., 2001). Second, hemodynamics is characterized by microvascular vasomotor responses and localized tissue oxygenation fluctuations, including prefrontal-motor cortical HbO2 changes and acupoint-level warm-needle hemodynamic responses (Si et al., 2021b; Ni et al., 2025). Third, biochemical metabolism is marked by transient adenosine release and other local molecular events linked to analgesic and anti-inflammatory signaling (Goldman et al., 2010; Chang et al., 2023). Fourth, electrophysiological activity is reflected in EEG periodic-aperiodic dynamics and Deqi-related cortical modulation (Yu et al., 2024a; 2024b).

Characterizing Deqi as the aggregate of these multidimensional states necessitates a departure from traditional biomeasurement strategies. Early digitalization efforts utilized rigid sensing modalities, including uniaxial dynamometers and silicon-based multiaxial modules. Such legacy systems exhibit mechanical impedance mismatch with soft biological tissues, causing signal distortion and attenuating practitioner tactile proprioception. To bypass these constraints, the field is moving toward flexible multimodal bioelectronics. Soft nanomesh pressure sensors and broader wearable-sensor design frameworks support conformal, low-interference acquisition of mechanical inputs while preserving clinical authenticity (Lee et al., 2020; Ates et al., 2022; Mahato et al., 2024).

High-fidelity mechanical decoding through flexible bioelectronics provides an empirical basis for examining the force-biology interface in acupuncture. The real-time mapping of tissue mechanics and biochemical fluctuations described above offers emerging evidence for mechanistic links between local cellular mechanosensation and central systemic reflexes. To evaluate how quantified physical stimuli cross the cell membrane barrier and influence downstream biology, we discuss the force-immune axis and neural circuits that contribute to systemic homeostasis.

The broad implementation of flexible arrays yields heterogeneous, multidimensional datasets that exceed the analytical thresholds of conventional linear statistics. Resolving such complexity requires clinically validated machine learning and multimodal AI frameworks that can integrate mechanical, optical, biochemical, electrophysiological and outcome data without overstating causal inference (Rajkomar et al., 2019; Soenksen et al., 2022; Chan et al., 2026; Chen et al., 2026). We evaluate multimodal Transformers and GNNs as candidate approaches for multirate temporal synchronization and spatiotemporal feature representation. The synthesis of these disparate sensory streams may enable a standardized, predictive Deqi Index, provided that external validation and explainability are incorporated into model development.

This acupuncture-specific trajectory is consistent with broader developments in digital health and bioelectronics. Recent reviews emphasize that wearable, ingestible, and implantable bioelectronics are moving from single-parameter monitoring toward integrated therapeutic and human-machine-interaction platforms. End-to-end wearable sensor design and hybrid multimodal wearable systems further highlight the need to combine mechanical, optical, electrochemical, and electrophysiological streams within robust data pipelines. Digital-biomarker reviews also show that passive sensor signals must be interpreted in relation to behavioral and psychosocial endpoints. For neurophysiological data, explainable AI reviews underscore the importance of transparent feature attribution, validation, and clinician-interpretable outputs when deep learning models are deployed in biomedical contexts (Ates et al., 2022; Mahato et al., 2024; Mishra et al., 2026; Presacan et al., 2026; Shah et al., 2026).

The Deqi microenvironment is defined as a dynamic, objective domain that integrates macroscopic physical, microscopic chemical, and electrophysiological processes. Flexible multimodal bioelectronic arrays can map four coupled dimensions of this domain *in situ*. First, tissue mechanics reflects viscoelastic remodeling of the collagen network and needle-rotation-induced winding effects that alter cytoskeletal tension. Second, hemodynamics captures microvascular perfusion changes and transient localized oxyhemoglobin (HbO2) fluctuations driven by autonomic vasomotor reflexes. Third, biochemical metabolism includes the local diffusion of signaling molecules, such as adenosine and serotonin, and downstream macrophage phenotype modulation. Fourth, electrophysiological signaling spans peripheral local twitch responses, sensory afferent discharges, and activation of systemic reflex circuits such as the vagal-adrenal pathway. Together, these measurable parameters reframe the historically subjective Deqi sensation as a quantifiable aggregate of local physical and biological responses.

## 2 Legacy rigid sensing: historical contributions and physical limitations

Prior to the advent of flexible electronics and nanomaterials, conventional rigid sensors served as indispensable precursors in the initial digitalization of acupuncture maneuvers. However, as the focus of clinical mechanism research shifts toward microscopic scales and *in-situ* monitoring, sensing equipment based on rigid silicon or traditional metallic elastomers has revealed substantial physical limitations that impede further scientific depth.

### 2.1 Evolution of mechanical quantification hardware

Initial quantification of acupuncture mechanics relied predominantly on modified uniaxial dynamometers, needle-handle force sensors, pressure sensors and displacement-tracking devices. Affixed typically to the needle handle or experimental stages, these instruments remained restricted to recording boundary forces or gross kinematic parameters, thereby failing to account fully for lateral shear dynamics arising from tissue anisotropy. Subsequent advancements in precision electromechanical engineering enabled the development of integrated multi-sensor platforms and robotic acquisition systems that record force, displacement, angle and manipulation trajectory for standardized acupuncture training and quantitative manipulation analysis (Gao et al., 2025; Liu J. et al., 2024; Li Y.-C. et al., 2025).

Recently, to mitigate the physical interference inherent in contact-based sensors, researchers have introduced tri-axial inertial sensors combined with computer vision, alongside non-contact high-frequency optical positioning systems such as OptiTrack. By attaching reflective markers to the practitioner’s hand or the needle hub, these technologies enable sub-millimeter 3D spatial trajectory reconstruction and dynamic modeling of complex compound maneuvers (Gao et al., 2025). Such six-axis torque sensors and optical navigation systems have provided useful tools for standardized acupuncture teaching and preservation of lineage-specific techniques, although they do not fully resolve tissue-level physiological measurement.

### 2.2 Mechanical dynamic distortion and proprioceptive interference

Despite the precise kinematic mapping provided by rigid multi-axis devices and external optical systems, their intrinsic physical and geometric attributes can alter the original mechanical characteristics of the needle and the authentic clinical environment. Traditional clamp-on sensors or electronic modules appended to the needle hub may increase the overall mass and shift the center of gravity, thereby changing the moment of inertia during rotational movements (Gao et al., 2025; Li Y.-C. et al., 2025).

Acupuncture is essentially a fine manual art heavily dependent on tactile feedback. Added mechanical load can disrupt the practitioner’s fingertip proprioception and tactile sensitivity during delicate adjustments. Non-contact optical navigation systems avoid direct added mass, but they still face practical challenges in complex clinical settings. Reflective markers may be occluded by the practitioner’s hands, clothing or the patient’s body, leading to frame loss and signal discontinuity. Moreover, camera frame rates and computational latency may limit the capture of high-frequency microtremors during rapid twirling or lifting-thrusting maneuvers (Zhang C. et al., 2025; Gao et al., 2025).

### 2.3 Mechanical mismatch and inflammatory artifacts

The most critical physical bottleneck for traditional rigid electronics lies in the mechanical mismatch between the device and biological soft tissues. Conventional silicon-based chips, rigid printed circuit boards and metallic modules usually have moduli far higher than those of skin, subcutaneous fascia and muscle. Reviews of wearable bioelectronics emphasize that reducing this modulus mismatch is essential for conformal contact, lower motion artifact and more stable long-term recordings at soft biological interfaces (Lee et al., 2020; Ates et al., 2022; Mahato et al., 2024).

This stiffness discrepancy prevents rigid sensors from maintaining conformal contact with irregular, highly elastic biological interfaces during needle-induced tissue deformation or respiratory fluctuations. It can introduce motion artifacts that are difficult to remove through post-processing, thereby reducing the signal-to-noise ratio of weak bioelectric signals such as sEMG or EEG. At the tissue interface, persistent relative sliding and friction may also impose unintended local shear loading, making it difficult to separate true acupuncture-evoked physiology from device-induced artifacts (Ates et al., 2022; Mahato et al., 2024).

### 2.4 The physics blind spot in tissue energy absorption

Conventional studies on rigid mechanical sensing frequently adhere to a paradigm of engineering reductionism, wherein data acquisition is predominantly restricted to “output force” (axial force or torque applied to the needle hub) or “input displacement” (insertion depth). However, this paradigm bypasses the evaluation of energy absorption and effective-work conversion from the perspective of *in vivo* tissue biomechanics and thermodynamics.

Living connective tissue is not an ideal linear elastic body; rather, it constitutes a nonlinear, viscoelastic and multilayered medium. Upon needle insertion into these structures, the total mechanical work exerted by the practitioner is not uniformly converted into the biological signals necessary to induce Deqi. A fraction of the mechanical energy is dissipated through viscous friction and interstitial fluid resistance, whereas the remaining energy is stored as effective elastic potential energy through structural remodeling of the three-dimensional collagen network. Connective-tissue winding models and shear-wave elastography studies support the importance of distinguishing applied input from tissue-absorbed effective mechanical work (Langevin et al., 2001; Koppenhaver et al., 2022).

It is specifically this “effective work,” precisely transferred to the target cell surfaces, that governs cytoskeletal tension redistribution, integrin clustering, and the subsequent biochemical cascades of mechanotransduction. Rigid force-sensing instrumentation, confined to measuring forces at the rigid-body boundary, lacks the capacity to quantitatively distinguish between total input energy and the effective work actually assimilated by the target fascial network. This limitation constitutes a profound blind spot in physics and energetic dynamics, particularly regarding the precise quantification of “tonifying” (Bu) and “reducing” (Xie) dosages in traditional acupuncture.

## 3 Decoding the mechanical stimulus: flexible bioelectronics for interference-reduced kinematic tracking

**FIGURE 1:**
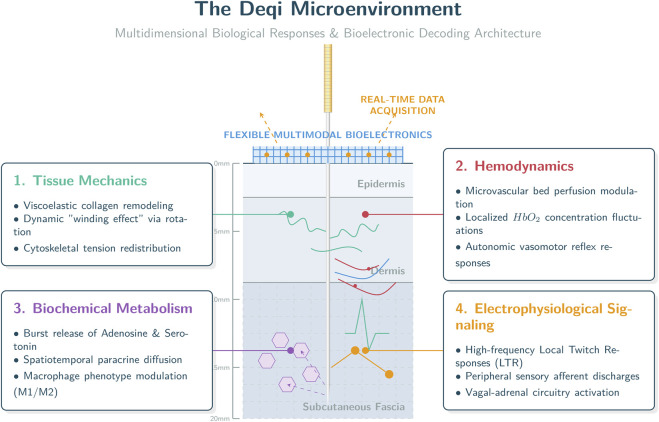
The multi-dimensional deqi microenvironment monitored by flexible bioelectronics.

Overcoming the mechanical constraints inherent in legacy rigid sensing necessitates the deployment of flexible bioelectronics for the high-fidelity deconstruction of the Deqi microenvironment. Through the emulation of skin-like stretchability, compliance and conformal contact at molecular and mesoscopic scales, these frontier materials facilitate low-interference integration with biological interfaces (Lee et al., 2020; Ates et al., 2022; Mahato et al., 2024). This technical evolution enables the interference-reduced decoding of needling kinematics while simultaneously establishing a multidimensional mapping framework to correlate mechanical inputs with systemic physiological outcomes (Figure 1).

**FIGURE 2:**
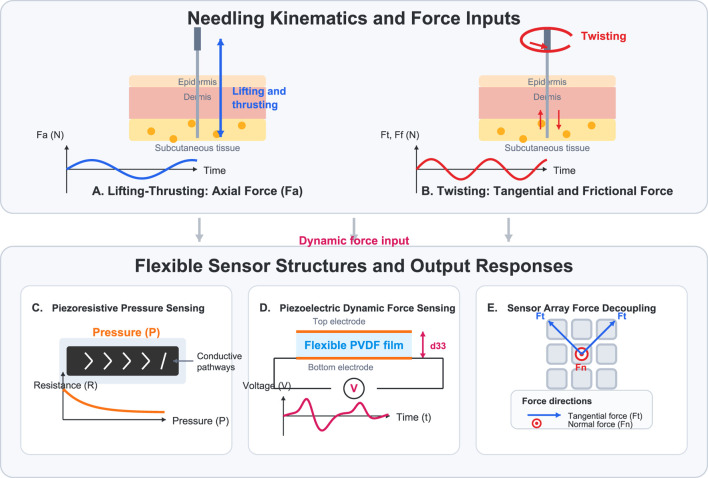
The conceptual and technical architecture of physical-biological signal mapping in smart acupuncture systems. **(A)** Lifting-thrusting manipulation and axial force input (Fa). **(B)** Twisting manipulation and tangential and frictional force inputs (Ft and Ff). **(C)** Piezoresistive pressure sensing. **(D)** Piezoelectric dynamic force sensing using a flexible PVDF film. **(E)** Sensor-array force decoupling of normal and tangential force components.

A structured hierarchy is illustrated, defining the pathway from innovative material fabrication to biological mechanism decoding. Figure 2 further summarizes the relationship between needling kinematics, force inputs, and flexible sensor output responses.

- The development of single-layer flexible electronic interfaces relies on fundamental materials science approaches aimed at establishing tactile-interference-free, soft interfaces, which integrate key technologies such as logic nanomesh, logic electrospun nanofibers, liquid metal circuits, and low-dimensional carbon nanomaterials to address the biomechanical mismatch at the needle-tissue junction.
- Integrated interfacing on dynamic tissue phantoms serves as a platform for demonstrating flexible sensing arrays in high-resolution spatial mapping. Advanced tissue phantoms and models enable high-fidelity quantification of dynamic needle-phantom interfaces, tissue biomechanics, and localized multidimensional responses during needle manipulation.
- Deciphering the biological code from peripheral to central systems represents a process in which quantified physical signals can be mapped to systemic biological effects. The diagram delineates the multi-scale mapping flow, from localized peripheral tissue microenvironments (mechanotransduction pathways and the force-immune axis) to central neural pathways (highlighting the PROKR2-dependent vagal-adrenal axis), thereby transforming subjective Deqi sensation into computable objective data.

### 3.1 Technical integration and breakthroughs in frontier sensing materials

The primary engineering challenge in constructing flexible sensing arrays involves resolving the “performance paradox,” namely, the requirement to combine large stretchability with high sensitivity (Gauge Factor, GF) under infinitesimal strains.

#### 3.1.1 Dynamic percolation networks of low-dimensional carbon

The inherent trade-off between macroscopic strain range and electromechanical sensitivity often limits the utility of mono-dimensional nanomaterials. Representative carbon-nanotube/elastomer systems illustrate this balance. Aligned CNT/TPU fibrous networks can sustain large strain and repeated cycling while retaining a response time on the order of tens of milliseconds (Ren et al., 2019). LIG-, CNT- and PU-based hybrid conductive networks similarly provide a plausible route for high-sensitivity finger-motion detection, but their reported gauge factors and strain ranges depend strongly on printed geometry, conductive-network architecture and readout electronics. Therefore, specific parameters should be reported with the original device source rather than transferred across sensor families (Wang et al., 2023).

#### 3.1.2 Liquid metals and MXene nanosheets

In regions requiring extreme conformality and subject to severe bending or torsion of the practitioner’s joints, room-temperature liquid metals such as Galinstan may serve as enabling materials for stretchable interconnects and deformable electrodes because of their metallic conductivity and fluidic deformability. However, acupuncture-specific validation remains limited, so these materials should be treated as candidate interconnect technologies rather than established Deqi sensors (Ates et al., 2022; Mahato et al., 2024). At the skin-device interface, MXene-based bioelectronic films have demonstrated low-impedance electrophysiological recording and stimulation capability; MXene-infused interfaces and broader flexible-electrode reviews support their relevance for weak bioelectric-signal acquisition, while oxidation, adhesion and long-term sweat exposure remain important limitations (Driscoll et al., 2021; Cheng et al., 2023).

### 3.2 “Invisible” monitoring via nanomesh and finger-sleeve sensors

To achieve objective quantification of acupuncture maneuvers without compromising the practitioner’s natural tactile perception, tactile-interference-free flexible electronic skin systems provide an important design precedent. Ultrathin nanomesh pressure sensors can monitor finger manipulation while minimizing sensory interference, supporting the principle that soft, breathable and conformal sensor architectures are preferable to bulky glove-like devices for manual needling applications (Lee et al., 2020; Ates et al., 2022).

#### 3.2.1 Ultra-thin nanomesh sensors

Electrospinning and nanomesh fabrication paradigms enable sub-micron, porous sensor architectures with high vapor transmission and low tactile burden. Driven by conformal contact, these micro-meshes can follow the epidermal topography of human fingertips and help preserve the mechanical signal fidelity required by dermal mechanoreceptors. Bypassing the tactile blunting and kinematic distortion inherent to conventional polymer gloves or rigid films, such architectures permit unobtrusive quantification of complex manual inputs, including pinch force, lifting-thrusting dynamics and twirling torque (Lee et al., 2020; Mahato et al., 2024).

#### 3.2.2 Pattern recognition in piezoelectric micro-arrays

Applied to the finger pulp, high-density piezoresistive or PVDF piezoelectric arrays, integrated with multi-axial torque calculation modules, facilitate the quantification of manipulation dynamics. High-frequency sampling, combined with sliding-window segmentation and dual-domain feature extraction, enables the resolution of subtle shear and normal force distributions during needling (Li Y.-C. et al., 2025). When implemented within lightweight machine learning frameworks, such as K-Nearest Neighbors, Support Vector Machines, or Random Forest, these systems achieve an overall 5-fold cross-validated recognition accuracy of 90.75%, with class-specific accuracies of 85.8% for “twirling-tonifying/reducing” and 94.2% for “lifting-thrusting-tonifying/reducing” maneuvers (Li Y.-C. et al., 2025). Consequently, these platforms offer a non-invasive digital strategy for the large-scale acquisition of master-level clinical heuristics and the subsequent parametric modeling of acupuncture protocols.

### 3.3 Critical technical challenges: nonlinear hysteresis mitigation and in vivo calibration

Notwithstanding the utility of flexible bioelectronics, significant materials engineering challenges remain in reconciling the high-frequency dynamic requirements of rapid lifting-thrusting with the high-precision static stability necessary for needle retention and *Deqi* maintenance.

Addressing nonlinear hysteresis is essential, as the inherent viscoelastic creep of polymer substrates, primarily polydimethylsiloxane (PDMS) and PU, coupled with the irreversible relative displacement of internal conductive filler networks under cyclic loading, can produce substantial hysteresis loops. Such phenomena distort kinematic waveforms during continuous manipulation and compromise signal fidelity. Representative flexible mechanical-sensing studies and reviews indicate that aligned conductive networks, ordered porous structures, pre-strain engineering and material-level calibration can improve dynamic stability, but hysteresis and baseline drift remain device-specific issues that must be reported explicitly (Ren et al., 2019; Wang et al., 2023).

In clinical environments, cutaneous sweat secretion and thermal fluctuations frequently alter capacitive dielectric constants and induce thermal impedance drift in piezoresistive modalities. Interface engineering strategies, such as superhydrophobic TiO2-ODI surface modification, have been reported to improve resistance to sweat- and humidity-induced interference while maintaining pressure sensitivity (Ma et al., 2023). Nevertheless, the absence of standardized performance evaluation frameworks, specifically regarding long-term dynamic linearity, perspiration resistance and batch-to-batch consistency, remains a significant barrier to scalable translation into clinical acupuncture practice (Ates et al., 2022; Mahato et al., 2024).

While decoding the kinematic actions of the practitioner precisely defines the mechanical “stimulus” (as discussed above), comprehensively understanding acupuncture demands an equal focus on the tissue’s “response”. The subsequent section shifts the perspective from the practitioner’s fingertips to the patient’s sub-dermal interface, exploring how multimodal flexible arrays capture the immediate physiological reactions—collectively termed the Deqi microenvironment.

## 4 Mapping the physiological response: multimodal sensing arrays for the in situ deqi microenvironment

While flexible input sensing addresses the kinematics of acupuncture (“how it is performed”), multimodal microenvironmental sensing elucidates the resulting physiological responses (“what it induces”). High-dimensional spatio-temporal mapping of the Deqi microenvironment marks a paradigm shift from qualitative subjective scales to objective biomarker topographies with high spatiotemporal resolution (Wu et al., 2026).

### 4.1 Tissue mechanics and in situ ultrasound-electric coupling

Acupuncture encompasses both neural electrical stimulation and a mechanical process that triggers extensive remodeling of localized connective tissue. As needles penetrate the subcutaneous fascia, rotation can induce mechanical entwining and coiling of collagen and elastin fibers, a winding effect that remains central to both traditional theory and contemporary connective-tissue biomechanics (Langevin et al., 2001). This localized mechanical deformation propagates through the fascial tension network. Real-time shear-wave elastography (SWE) provides dynamic quantification of this process. Randomized trials using SWE have demonstrated a marked reduction in the shear modulus of the erector spinae following dry needling in patients with chronic low back pain. Such data offer biomechanical support for muscle-tension release associated with needling responses (Koppenhaver et al., 2022).

Furthermore, high-frequency ultrasonic imaging enables the precise delineation of effective versus hazardous depths across anatomical strata, establishing cross-sectional area and muscle thickness as dynamic imaging biomarkers for acupuncture-induced morphological alterations during muscle rehabilitation. To address acoustic shadowing from conventional metallic or thick polymer electrodes, ultra-thin, ultrasound-transparent high-density electromyography interfaces, such as the ReGrid system, have been developed (Cerone et al., 2026). These devices use conformable, acoustically transparent electrode architectures to enable simultaneous ultrasound imaging and HD-sEMG detection. This coupling helps link deep-tissue geometric deformation with superficial muscle activation potentials during needling.

### 4.2 *In vivo* real-time mapping of hemodynamics and biochemical metabolic networks

The occurrence of Deqi is frequently accompanied by profound fluctuations in blood perfusion within the local microcirculatory network and the burst release of metabolic markers with analgesic and anti-inflammatory properties. The introduction of flexible optical systems and micro-scale chemical sensors allows these transient *in vivo* biochemical events to be captured continuously and in real-time.

While traditional photoplethysmography (PPG) is limited to detecting superficial capillary blood flow, multi-channel functional near-infrared spectroscopy (fNIRS) enables the assessment of oxygenation dynamics deep within tissue and cortex. Brain science research indicates that acupuncture-induced Deqi sensations are associated with decreased HbO2 concentrations within the bilateral prefrontal cortex and motor cortex, suggesting that potent peripheral mechanical stimuli may reorganize cognitive and pain-related cortical networks through blood-flow modulation and functional-connectivity changes (Si et al., 2021b). At the peripheral acupoint, customized NIRS probes show that warm-needle moxibustion can elevate local HbO2 and tissue oxygen saturation compared with mechanical acupuncture alone, providing optical evidence for local microcirculatory modulation (Ni et al., 2025).

Frontier bioelectronic technologies enable the *in situ* conversion of conventional acupuncture needles into multifunctional electrochemical and optical molecular microprobes. Microelectrochemical smart needles have been developed for real-time, minimally invasive monitoring of tissue oxygenation-related electrochemical signals (Vieira et al., 2020). SERS needle probes based on acupuncture-needle technology have also been used for non-destructive quantification of subarachnoid-hemorrhage-related microRNAs in cerebrospinal fluid, with detection limits reported in the femtomolar range (Sun et al., 2022). In parallel, surface molecularly imprinted nanocavity/CNT-functionalized acupuncture needle microelectrodes illustrate the feasibility of MIP-modified needle sensors for small-molecule detection (Li Q. et al., 2025). For acupuncture-specific biochemical dynamics, flexible needle-type microbiosensors have enabled real-time *in vivo* monitoring of acupuncture-mediated adenosine release (Chang et al., 2023).

### 4.3 Electrophysiological microenvironment and fractal brain dynamics

Electrophysiological signals serve not merely as byproducts of acupuncture but as the primary carriers for the transmission of peripheral mechanical information to the central nervous system.

Peripheral high-density electromyography and local twitch response (LTR) capture are facilitated by low-impedance, conformal flexible electrophysiological interfaces, including MXene-based or other skin-conformal electrode architectures. These interfaces allow researchers to record synchronized peripheral nerve and muscle discharges during needle insertion with high spatiotemporal resolution. Their dynamic adherence can reduce motion artifacts and polarization-related interference compared with rigid gel electrodes, thereby improving the extraction of weak acupuncture-evoked electrical signals (Driscoll et al., 2021; Cheng et al., 2023; Cerone et al., 2026).

Contemporary investigations into central aperiodic EEG dynamics have moved beyond traditional analyses focused only on isolated frequency bands, such as absolute Alpha and Theta power. Recent acupuncture studies using periodic-aperiodic EEG measurements have introduced aperiodic or fractal signal analysis to evaluate acupuncture mechanisms. These findings suggest that peripheral neural stimulation and acupuncture efficacy may be accompanied by measurable changes in the aperiodic EEG slope (1/f slope) within sensorimotor and prefrontal cortices (Yu et al., 2024a; 2024b).

Within computational neuroscience, fluctuations in the slope of fractal dynamics can serve as objective metrics for shifts in the cortical excitation/inhibition (E/I) balance. When synthesized with EEG microstate spatial clustering, which provides millisecond-scale temporal resolution, evidence indicates that Deqi sensations modulate transition probabilities and dwelling durations of specific EEG microstate topographies. These findings clarify immediate central mechanisms through which the Deqi phenomenon may regulate attentional allocation and affective components of pain perception (Si et al., 2021a; Yu et al., 2024a; 2024b).

To complement the qualitative comparison presented in Table 1, the representative sensing platforms discussed in this review are quantitatively compared in Table 2. The comparison encompasses spatial and temporal resolution, sampling frequency, sensitivity or detection limit, response time, operating range, and durability. Because these platforms measure fundamentally different physical, electrophysiological, optical, and biochemical variables, their performance metrics are not directly interchangeable. Nevertheless, this comparison clarifies their respective suitability for capturing needling kinematics and the multidimensional Deqi microenvironment. The correspondence between sensing modalities, measurable biomarkers, and their physiological and clinical interpretation is further summarized in Table 3.

**TABLE 1:** Multidimensional comparison between legacy rigid sensing and flexible bioelectronics.

Assessment dimension	Legacy rigid sensing and positioning systems	Flexible bioelectronics and conformal sensing systems	Core discrepancies in clinical and mechanistic insights
Young’s modulus matching	Extreme mismatch (10–100 GPa vs. tissue 10–100 kPa)	High conformal matching (10 kPa–1 MPa, conformally adhering to skin texture)	Rigid devices tend to trigger microscopic interface friction, abnormal Piezo channel activation, and immune rejection; flexible devices maintain natural microenvironment homeostasis
Proprioceptive and tactile interference	Significant interference (substantially alters the needle’s center of gravity, mass, and moment of inertia)	Near-perceptual monitoring (electrospun nanomeshes, breathable without shielding tactile corpuscles)	Flexible devices may support the reproduction of subtle ‘flying-needle-and-qi-movement’ (Feijing Zouqi) signatures and capture relevant maneuver signals under interference-reduced conditions
Physical signal acquisition perspective	Isolated, unidirectional boundary acquisition (capturing only externally applied displacement or mechanical force)	Bidirectional mechanical interaction sensing (simultaneously capturing practitioner’s force, tissue reaction force, and local strain)	Flexible arrays, combined with rheological models, calculate ‘effective absorbed work,’ providing energetic support for quantifying the mechanical dosage of ‘tonifying’ (Bu) and ‘reducing’ (Xie) maneuvers
Signal purity and resolution	Susceptible to severe broadband motion artifacts	Dynamic followership and adherence effectively suppress relative slip, resulting in high SNR (low impedance)	Flexible devices can extract ultra-low-frequency slow waves or high-frequency weak physiological signals LTR that conventional electrodes cannot capture

**TABLE 2:** Quantitative performance comparison of representative sensing platforms for decoding needling kinematics and the Deqi microenvironment.

Sensing platform	Primary target relevant to deqi	Spatial resolution	Temporal resolution/Sampling frequency	Sensitivity or detection limit	Response time	Durability/Operating range	Principal strengths and limitations	Representative references
Rigid uniaxial or multi-axis force/torque sensors	Axial force, lateral force, torque, insertion displacement and externally applied mechanical work	Point measurement at the needle handle or instrument boundary; unable to spatially map tissue strain	Approximately 100 Hz for representative robotic torque-feedback streams; device-dependent	Sensor-specific; not consistently reported in acupuncture studies	System-dependent; NR	Generally reusable with high mechanical durability	High accuracy for boundary force measurement, but added mass changes the needle’s center of gravity and moment of inertia; cannot distinguish total input work from tissue-absorbed effective work	Gao et al., 2025; Li et al., 2025b
Optical motion-capture systems and inertial measurement units	Three-dimensional needle trajectory, lifting-thrusting amplitude, rotation angle and manipulation velocity	Sub-millimeter three-dimensional positioning under unobstructed conditions	Camera- and IMU-dependent; conventional systems may inadequately capture microtremors exceeding 100 Hz	NA for force or biochemical measurements	Frame acquisition and reconstruction latency; NR	Reusable; no direct mechanical fatigue at the skin interface	Non-contact trajectory reconstruction with minimal added mechanical load, but susceptible to marker occlusion, frame loss and discontinuous data	Gao et al., 2025; Zhang et al., 2025a
Aligned CNT/TPU fibrous strain sensors	Finger deformation, subtle hand motion and manipulation-related strain	Determined by sensor dimensions and array pitch; localized surface-strain measurement	Response time approximately 70 m; sampling frequency determined by readout electronics	Minimum detectable strain approximately 0.5%	Approximately 70 m	Working strain up to approximately 900%; stable over approximately 10,000 cycles at 200% strain	Wide strain range and high cyclic durability; potential limitations include viscoelastic hysteresis, baseline drift and indirect estimation of needle force	Ren et al. (2019)
CNT/LIG/PU hybrid piezoresistive strain sensors	Fine finger motion, lifting-thrusting dynamics, twirling and low-amplitude muscular vibration	Device- and array-geometry-dependent	Millisecond-scale dynamic response; exact sampling frequency NR	Gauge factor and strain range are device-specific; values must be verified from the original device source	Millisecond scale	Cyclic durability NR in the cited acupuncture-oriented discussion	Combines high sensitivity with large deformation tolerance, but conductive-network rearrangement can cause nonlinear hysteresis and batch variability	Wang et al., 2023 ; original device source should be verified
Superhydrophobic TiO2-ODI-modified flexible pressure sensors	Normal pressure, fingertip contact force and local pressure distribution	Array-pitch-dependent localized pressure mapping	High-frequency dynamic monitoring; sampling frequency NR	Approximately 0.83 kPa–1	Millisecond scale	Enhanced resistance to sweat and moisture; cycle number NR	Reduced perspiration-induced interference and improved interface stability; temperature drift and long-term calibration remain unresolved	Ma et al. (2023)
PVDF/PU nanomesh and piezoelectric micro-arrays	Pinch force, normal and shear forces, twirling torque and high-frequency microvibration	Sub-micron fibre thickness; functional spatial resolution determined by electrode pitch rather than fibre diameter	Suitable for high-frequency transient events; exact sampling frequency NR	Device-dependent; NR	Rapid piezoelectric response, generally within the millisecond range	Repeated-bending durability and long-term signal stability remain insufficiently standardized	Ultralight, breathable and minimally disruptive to tactile perception; piezoelectric systems are less suitable for static or slowly varying force because of charge leakage	Lee et al., 2020; Mahato et al., 2024
MXene- or LIG-based flexible HD-sEMG arrays	Local twitch responses, muscle activation, peripheral electrophysiological discharge and motor-unit recruitment	High-density spatial mapping; electrode spacing and channel density are device-dependent	Approximately 1,000–2,000 Hz for representative HD-sEMG acquisition	Skin-electrode impedance can be low; exact impedance is device- and interface-dependent	Millisecond-scale electrophysiological response	Reusable over repeated recording sessions; long-term oxidation, sweat exposure and adhesion durability require further evaluation	Low impedance and conformal contact improve weak-signal SNR and reduce motion artifacts; oxidation, perspiration and cross-channel interference remain concerns	Driscoll et al., 2021; Cheng et al., 2023; Cerone et al., 2026
Shear-wave elastography and high-frequency ultrasound combined with ultrasound-transparent HD-sEMG patches	Fascial winding, shear modulus, muscle thickness, cross-sectional area and coupled muscle electrical activity	Anatomical-layer-resolved imaging; probe-frequency-dependent	Real-time imaging; frame rate is device-dependent and NR in the reviewed acupuncture studies	Tissue stiffness quantified in kPa; ultrasound transparency reported as >95% for representative conformal electrode systems	Near-real-time imaging and electrophysiological acquisition	Imaging probes are reusable; patch adhesion and repeated-use durability are device-dependent	Simultaneous structural and electrophysiological assessment supports mechanoelectrical coupling analysis; limitations include operator dependence, probe pressure and acoustic shadowing	Koppenhaver et al., 2022; Cerone et al., 2026
Flexible PPG and multichannel fNIRS systems	Local perfusion, HbO2, deoxyhemoglobin, total hemoglobin and tissue oxygen saturation	Regional mapping determined by source-detector spacing; lower spatial resolution than ultrasound or HD-sEMG	Approximately 10 Hz for representative fNIRS streams; physiological hemodynamic responses occur over seconds	No universal LOD; sensitivity depends on optical path length, wavelength and tissue properties	Seconds because of neurovascular and microvascular response delay	Reusable; sensitive to probe displacement, ambient light and optical-coupling changes	Enables continuous non-invasive hemodynamic monitoring, but has limited depth specificity, relatively slow physiological response and susceptibility to systemic vascular confounders	Si et al., 2021b; Ni et al., 2025
Flexible needle-type electrochemical adenosine microsensors	Local adenosine release associated with acupuncture analgesia and mechanical-dose response	Highly localized measurement around the needle sensing region	Continuous, second-scale monitoring	Linear range approximately 0–50 uM; sensitivity approximately 1.1 nA uM-1	<5 s	Good short-term reproducibility and selectivity; chronic implantation durability NR	Directly captures rapid biochemical events at the acupoint; susceptible to biofouling, enzyme degradation, calibration drift and tissue-response artifacts	Chang et al. (2023)
Needle-based molecularly imprinted polymer sensors	Trace metabolites, proteins or inflammatory biomarkers in local interstitial fluid	Localized needle-tip or needle-surface measurement	Platform-dependent; second-scale monitoring is theoretically achievable but not uniformly reported	Representative LOD is analyte- and matrix-dependent; values should be verified from the original source	NR	Molecular-imprinting layers can support repeated recognition, but *in vivo* regeneration cycles and long-term stability are NR	High molecular selectivity and minimally invasive sampling; limitations include slow mass transport, nonspecific adsorption and matrix-dependent calibration	Li et al. (2025c)
Gold-nanostructure SERS needle probes	miRNAs and other low-abundance molecular biomarkers	Localized optical interrogation around the functionalized needle tip; laser-spot-dependent	Acquisition-time-dependent; not generally suitable for continuous high-frequency monitoring	Reported LOD as low as approximately 0.1 fM for representative miRNA detection	NR; commonly dependent on spectral integration and preprocessing time	Substrate reproducibility and repeated-use stability remain incompletely established	Extremely low molecular detection limits and molecular fingerprinting capability; major barriers include signal variability, optical instrumentation requirements and limited continuous-monitoring capacity	Sun et al., 2022; Zhou et al., 2018

Abbreviations: CNT, carbon nanotube; HD-sEMG, high-density surface electromyography; IMU, inertial measurement unit; LIG, laser-induced graphene; LOD, limit of detection; MXene, transition-metal carbide/nitride; NA, not applicable; NR, not reported; PPG, photoplethysmography; PU, polyurethane; PVDF, poly(vinylidene fluoride); SERS, surface-enhanced Raman scattering; SWE, shear-wave elastography; TPU, thermoplastic polyurethane.

Values are representative device-level metrics rather than universal specifications. NR, not reported; NA, not applicable. Sensitivity, impedance and LOD, describe different physical quantities and should not be directly ranked.

**TABLE 3:** Biomarker mapping system of the deqi microenvironment empowered by multimodal flexible sensing technologies.

Physical/Chemical dimensions of microenvironment	Frontier flexible sensors and advanced analytical technologies	Key target biomarkers/Extracted physical quantities	Insights into physiological mechanisms and clinical evaluation significance
Biomechanical Deformation	SWE, Flexible Piezoresistive Strain Arrays, ReGrid Ultrasound-Transparent Patches	Young’s modulus (kPa), Muscle CSA, Deformation Rate of Deep Fascial Fibers	Dynamically quantifies the “winding effect” of connective tissues and the elastic potential energy released by mechanical traction, allowing *in situ* observation of tissue stiffness resolution
Hemodynamics and Microcirculation	Flexible Highly Conformal PPG Patches, Multi-Channel fNIRS Dual-Layer (Brain/Muscle) Oximetry Devices	HbO2 Concentration, Local Tissue Oxygen Saturation (StO2) Fluctuations	Characterizes PFC network reorganization during Deqi and provides supporting evidence for warm-needle effects on local microcirculation
Biochemical and Molecular Metabolism	Needle-Based MIP (Molecularly Imprinted Polymer) Microelectrodes, Needle-Type SERS Probes, Magnetically Responsive Nanomeshes	Adenosine, 5-HT, Endorphins, miRNA-21-5p	Bypasses the latency of conventional blood sampling, achieving second-scale quantitative molecular probing at single-cell and *in vivo* minimally invasive levels, thereby delineating the dose-response relationship of mechanical stimuli
Neuroelectrophysiological Feedback	MXene-Based Low-Impedance HD-sEMG Patches, Aperiodic EEG Analysis, EEG Microstate Mapping	Local Twitch Response (LTR) Frequency, Flattening Degree of EEG 1/f Slope, Transition Probabilities of EEG Microstates	Globally maps the cross-scale spatiotemporal dynamic cascade network from local neuronal activation to whole-brain E/I homeostatic reconstruction

In summary, the transition from qualitative clinical observation to quantitative digital mapping depends on high-spatiotemporal-resolution data streams provided by flexible sensing arrays. These multimodal platforms serve as biophysical measurement tools that capture transient *in situ* dynamics of the Deqi microenvironment, ranging from fascial strain to paracrine signaling cascades. However, objective quantification of these physical and chemical biomarkers also requires biological interpretation. By linking captured sensor data with systemic physiological responses, the following section delineates cellular mechanotransduction pathways and neural circuits that may transform localized mechanical inputs into measurable therapeutic outcomes.

Multimodal sensing arrays provide high-resolution spatiotemporal phenotypes of the Deqi microenvironment, but they primarily represent outward physical and chemical manifestations of acupuncture. To understand why these microenvironmental shifts occur, the biological mechanisms must also be considered. The following section outlines mechanotransduction pathways that connect externally mapped physical forces with internal systemic biological responses.

## 5 Bridging physics and biology: decoding the cellular and neural cascades of mechanotransduction

High-fidelity mechanical decoding through flexible bioelectronics provides an empirical basis for examining the force-biology interface in acupuncture. The real-time mapping of tissue mechanics and biochemical fluctuations described above offers emerging evidence for mechanistic links between local cellular mechanosensation and central systemic reflexes. To evaluate how quantified physical stimuli cross the cell membrane barrier and influence downstream biology, we discuss the force-immune axis and neural circuits that contribute to systemic homeostasis.

### 5.1 Telocytes and the peripheral force-immune axis

The mechanical reconfiguration of the three-dimensional collagen network, which is macroscopicly captured *in situ* by the ultrasound-transparent sensing patches, provides the necessary physical traction to activate mechanosensitive Ptgs2+ telocytes.

Early mechanotransduction models in acupuncture localized the initial sensory response to dermal fibroblast deformation and pressure-induced mast cell degranulation. Recent structural and functional analyses integrate advanced immunofluorescence, computational morphological recognition and mechanistic validation to identify a distinct interstitial sensor population (Zhang Z. et al., 2025). These cells, designated as Ptgs2+ connective-tissue proximal telocytes, densely populate deep connective tissue matrices such as the fascia of the linea alba.

Telocytes project elongated cytoplasmic extensions termed telopodes. These specialized structures span tens to hundreds of micrometers. The dense interweaving of telopodes throughout the ECM generates a continuous three-dimensional network. This specific geometric architecture translates macroscopic tissue deformation into localized cellular mechanical stress and matrix strain.

The mechanical reconfiguration of the three-dimensional collagen network, captured *in situ* by ultrasound-transparent sensing patches, provides the necessary physical traction to activate mechanosensitive Ptgs2+ telocytes. This suggests that the tissue stiffness resolution observed via shear wave elastography is the macroscopic manifestation of microscopic cytoskeletal tension redistribution within the fascial matrix.

Single-cell transcriptomics isolate a highly mechanosensitive telocyte subpopulation defined as Ptgs2+ CPTCs (Cd34+/Pdgfra+). Specific needle maneuvers generate targeted physical traction at localized acupoints. This applied mechanical input directly modulates the telocyte lineage. Following intervention, the local Ptgs2+ CPTC population undergoes rapid cellular expansion. These mechanically stimulated interstitial cells subsequently demonstrate fortified intercellular adhesion and upregulated biochemical signaling connectivity.

Needle-type multi-enzyme/Prussian-blue microbiosensors have been used to monitor acupuncture-mediated adenosine release *in vivo*, supporting real-time characterization of local purinergic biochemical dynamics rather than direct inference of macrophage polarization (Chang et al., 2023). Separately, evidence for Ptgs2+ connective-tissue proximal telocytes and M2 macrophage polarization comes from mechanistic cellular and immunological analyses, which should be interpreted as a distinct force-immune pathway rather than as a result confirmed by adenosine sensing alone (Zhang Z. et al., 2025).

More significantly, *in vivo* and *in vitro* mechanistic validations have confirmed that mechanosensitive Ptgs2+ CPTCs activate the Il6-Il6st-Stat3 intracellular signaling pathway via cytokine secretion. This process drives the polarization of resident macrophages toward an M2 phenotype characterized by anti-inflammatory and tissue-repair functions (Zhang Z. et al., 2025). This cytological discovery supports the Force-Immune Axis triggered by acupuncture and positions Ptgs2+ telocytes as an important hub linking mechanical stimulation to immune modulation.

Beyond the peripheral tissue, electroacupuncture (EA) at specific frequencies demonstrates a capacity for immune remodeling across central neuroimmune pathways. Evidence indicates that acupuncture can regulate microglial polarization through alpha7 nicotinic acetylcholine receptor-mediated autophagy, shifting microglia from pro-inflammatory M1-like states toward more reparative phenotypes (Dong et al., 2026). This central immune-modulatory pathway provides a mechanistic link between peripheral stimulation, neuroinflammation, and pain modulation.

### 5.2 Neural circuit specificity and prefrontal projections

The neural action potentials generated locally by acupuncture do not propagate in a disordered or diffuse manner within the spinal cord. Instead, they rely on highly specific, evolutionarily conserved central neural circuits to induce precise systemic functional regulation of internal organs.

In the context of the body-brain anti-inflammatory and anti-epileptic vagal circuit, acupuncture has long been used for complex systemic immune and neurological disorders (Wang et al., 2026a). The field has historically lacked a precise neuroanatomical basis for acupoint specificity. A Nature study demonstrated that low-intensity EA at specific distal hindlimb acupoints activates PROKR2-Cre-expressing sensory neurons that innervate deep hindlimb fascia rather than abdominal fascia (Liu et al., 2021). These afferent signals recruit brainstem circuits that drive the vagal-adrenal axis and trigger catecholamine release from the adrenal medulla, thereby supporting systemic anti-inflammatory effects. This molecular and circuit-level evidence provides modern neuroanatomical support for acupoint specificity. Localized HbO2 fluctuations in the prefrontal cortex and flattening of the aperiodic EEG slope may serve as measurable digital biomarkers of these deeper autonomic reflex arcs.

In the context of vascular cognitive impairment (VCI), a condition prevalent in geriatric populations, circuit-level investigations indicate that EA at discrete frequencies can modulate deep neural circuitry within the LC-PFC axis (Wan et al., 2025). The resulting elevation of norepinephrine (NE) levels in the PFC enhances synchronous firing within cognitive neuronal ensembles while simultaneously augmenting microvascular perfusion, quantified as cerebral blood flow (CBF). This mechanism facilitates the reversal of working memory deficits in animal models (Wan et al., 2025). Furthermore, dynamic functional connectivity data indicate that acupuncture can reorganize limbic-paralimbic-neocortical networks during stimulation, providing complementary evidence that Deqi-related afferent input engages distributed central networks rather than a single isolated cortical site (Wang Z. et al., 2026).

To make the biological logic of Deqi more explicit, the major mechanotransduction pathways are summarized in Table 4 according to initiating stimulus, cellular sensor, downstream signaling, physiological outcome and measurable sensor biomarkers.

**TABLE 4:** Mechanotransduction pathways linking acupuncture stimuli to measurable Deqi-related biomarkers.

Mechanotransduction pathway	Initiating stimulus	Cellular sensor	Downstream signaling	Physiological outcome	Supporting evidence	Measurable sensor biomarkers
Connective-tissue winding and fascial tension propagation	Needle rotation, lifting-thrusting and local shear	Fibroblasts, telocytes and mechanosensitive connective-tissue networks	Cytoskeletal remodeling, ECM tension redistribution and mechanosensitive ion-channel activation	Local stiffness change, fascial release and altered afferent input	Connective-tissue mechanism and SWE dry-needling evidence (Langevin et al., 2001; Koppenhaver et al., 2022)	SWE shear modulus, ultrasound CSA/MMT, force/torque, strain maps
Ptgs2+ telocyte force-immune axis	Mechanical deformation of subcutaneous fascia	Ptgs2+ CPTCs/telocytes	IL-6-IL6ST-STAT3 signaling and macrophage polarization	Anti-inflammatory M2-like response and tissue repair	Force-immune axis evidence in acupuncture (Zhang et al., 2025c)	Local stiffness, cytokine panels, immune-cell phenotype markers, adenosine dynamics
Purinergic adenosine analgesic pathway	Needle manipulation and local ATP/adenosine release	Purinergic receptors on nociceptive afferents and local cells	Adenosine A1 receptor signaling and local neuromodulation	Analgesia and mechanical-dose response	Adenosine A1 mechanism and needle-type adenosine biosensing (Goldman et al., 2010; Chang et al., 2023)	Needle-type adenosine signal, pain score, local biochemical time series
PROKR2-dependent vagal-adrenal reflex	Low-intensity EA at deep hindlimb acupoints	PROKR2+ sensory afferent neurons innervating deep fascia	Brainstem recruitment and vagal-adrenal catecholamine release	Systemic anti-inflammatory effect and acupoint specificity	Neuroanatomical evidence for EA-driven vagal-adrenal axis (Liu et al., 2021)	fNIRS HbO2, autonomic indices, inflammatory cytokines, stimulation-site specificity
alpha7nAChR-mediated microglial polarization	Peripheral EA and central neuroimmune modulation	Microglia expressing alpha7 nicotinic acetylcholine receptors	Autophagy activation and M1-to-M2 phenotypic conversion	Reduced neuroinflammation and inflammatory pain	Acupuncture-regulated microglial polarization through alpha7nAChR-mediated autophagy (Dong et al., 2026)	EEG/fNIRS neurophysiology, inflammatory cytokines, pain-related behavioral readouts
LC-PFC neurovascular modulation	Frequency-specific EA in vascular cognitive impairment models	LC noradrenergic neurons and prefrontal cortical networks	Norepinephrine release, cerebral blood-flow modulation and network synchronization	Improved working memory and cortical perfusion	EA modulation of LC-PFC circuit and cerebral blood flow (Wan et al., 2025)	fNIRS HbO2, CBF, EEG dynamics, cognitive outcome measures

The pathways are organized as a mechanistic map rather than as mutually exclusive modules. A single acupuncture stimulus may activate several pathways simultaneously, and the listed biomarkers represent measurable proxies rather than complete causal readouts.

## 6 Artificial intelligence empowerment: multimodal data fusion and the construction of the “Deqi Index”

The deployment of flexible multimodal bioelectronic arrays across laboratory and clinical studies is producing heterogeneous, high-dimensional datasets in acupuncture research (Ates et al., 2022; Mahato et al., 2024). Recent reviews of AI-assisted acupuncture indicate that machine learning has been applied mainly to efficacy prediction, manipulation recognition, prescription recommendation, acupoint sensitization analysis and robotic acupoint localization (Chan et al., 2026; Chen et al., 2026). Because these datasets combine mechanical waveforms, hemodynamic signals, electrophysiological recordings, biochemical measurements and clinical outcomes, conventional linear statistics may not fully capture nonlinear cross-modal interactions.

### 6.1 Multirate temporal synchronization for heterogeneous data alignment

To evaluate a localized Deqi window, AI systems must concurrently process heterogeneous streams such as multidimensional torque feedback from robotic systems, HD-sEMG signals, fNIRS hemodynamic signals and needle-based biochemical measurements. These streams differ in sampling rate, latency, amplitude scale and noise structure, creating challenges in synchronization, phase alignment and dimensionality reduction (Li Y.-C. et al., 2025; Cheng et al., 2023; Si et al., 2021b; Chang et al., 2023). A robust AI architecture should therefore perform temporal alignment and quality control before downstream feature fusion or prediction.

Frontier architectures for multimodal medical signal processing have introduced adaptive alignment and feature-fusion strategies for heterogeneous clinical and wearable data. In the setting of acupuncture, these approaches should be described cautiously because direct evidence for validated cross-modal Transformer models remains limited. Broader multimodal AI and wearable-sensor reviews indicate that attention-based fusion may help integrate asynchronous mechanical, optical, biochemical, and electrophysiological signals, but task-specific validation is required before such models can be treated as established acupuncture tools (Soenksen et al., 2022; Mahato et al., 2024; Presacan et al., 2026).

### 6.2 Evolution of algorithmic paradigms: From SVM to transformers

The objective of AI-supported acupuncture evaluation has shifted from static feature classification toward spatiotemporal representation learning, although the current evidence base remains early and methodologically heterogeneous (Chan et al., 2026; Chen et al., 2026). A comparative overview of the principal AI approaches, including their input data, typical tasks, advantages, limitations, and validation strategies, is provided in Table 5.

**TABLE 5:** Comparison of AI approaches for multimodal acupuncture and Deqi-related data analysis.

AI approach	Input data	Typical task	Advantages	Limitations	Validation strategy
SVM	Handcrafted Deqi questionnaire features, routine clinical variables, time-domain force or motion features	Responder prediction and basic manipulation classification	Works with small datasets; interpretable decision boundary when feature set is limited	Depends strongly on manual feature engineering; limited temporal-context modeling	10-fold CV or internal train-test split in reported FD prediction studies (Chen et al., 2022b; Yin et al., 2022)
Random forest/XGBoost	Routine clinical variables, biomarker panels or network-topology features	Feature ranking, prognosis stratification and exploratory mechanism mining	Captures nonlinear interactions and provides variable importance	May overfit small single-center datasets; causal interpretation is limited	Internal validation, cross-validation and prospective external validation when available (Li et al., 2025a)
CNN-LSTM	Video frames, IMU trajectories, force waveforms or synchronized maneuver sequences	Needling maneuver recognition and training assessment	CNN extracts local spatial patterns while LSTM captures temporal order	Requires annotated sequences; performance can degrade across operators or sites	Hold-out, k-fold or operator-level validation depending on dataset design (Tu et al., 2021; Zhu et al., 2023)
GNN	EEG/fNIRS connectivity graphs, multimodal sensor networks or anatomical sensor adjacency	Brain-state representation, graph-level classification and topology-aware fusion	Models relational structure among channels, sensors or anatomical nodes	Graph construction assumptions can dominate results; external validation remains limited	Graph-level cross-validation, subject-level split or LOSO validation when sample size permits
Transformer	Long multichannel time series, video-sensor sequences, clinical records and multimodal feature tokens	Cross-modal fusion, sequence modeling and long-range dependency learning	Attention can model asynchronous and long-range interactions across modalities	Data-hungry; susceptible to spurious attention patterns; acupuncture-specific evidence is still early	Predefined train/validation/test split, external cohort testing and ablation of modalities
XAI	Model outputs, sensor features, saliency maps, SHAP values or attention explanations	Clinical interpretation, biomarker attribution and safety review	Improves transparency and helps identify clinically plausible biomarkers	Explanations may be unstable or method-dependent; does not prove causality	Explanation consistency checks, clinician review, sensitivity analysis and reporting standards (Liu et al., 2020; Presacan et al., 2026)

Validation terminology should be stated explicitly in future studies. K-fold cross-validation, leave-one-subject-out validation and external cohort testing answer different questions and should not be conflated.

Within classical machine learning and spatiotemporal hybrid deep networks, early investigations relied extensively on algorithms such as support vector machines (SVM) and random forest. For instance, by manually extracting time-domain features, such as peak values, variance, and zero-crossing rates, from segmented mechanical waveforms of acupuncture maneuvers, SVM algorithms reported an overall 5-fold cross-validated accuracy of 90.75%, with 94.2% for lifting-thrusting tonifying/reducing and 85.8% for twirling tonifying/reducing (Li Y.-C. et al., 2025). Feature engineering for physiological time series can also include frequency-domain descriptors and nonlinear recurrence- or complexity-based features; however, these feature classes have been less consistently reported in the currently cited acupuncture-manipulation recognition studies than time-domain kinematic descriptors and video-based spatiotemporal representations (Chan et al., 2026; Chen et al., 2026). Classical models remain dependent on manual feature engineering and, without explicit memory units, have limited ability to process long-term contextual dependencies within compound maneuvers. Hybrid 3D CNN-LSTM architectures can capture implicit spatiotemporal features in high-frequency clinical video streams and support automated scoring against expert benchmark datasets (Tu et al., 2021; Zhu et al., 2023).

In the context of graph-based and manifold-learning analysis, traditional frequency-domain power analysis is insufficient for characterizing whole-brain EEG functional connectivity induced by acupuncture. A study of acupuncture-related brain functional networks used ISOMAP manifold learning to identify low-dimensional neural manifolds from high-dimensional EEG connectivity features (Li et al., 2022). Building on this principle, graph convolutional networks and spatio-temporal graph models may help learn data-driven interactions among multivariate physiological time series. Integrated attention modules can also highlight critical intervals during which physiological homeostasis changes, improving interpretability when appropriate validation data are available.

Driven by the rise of multimodal medical models and Transformer architectures, biomedical engineering has begun incorporating self-attention mechanisms into wearable sensor data processing. For acupuncture manipulation recognition, the current evidence base is stronger for sensor/video-based classification systems than for validated acupuncture-specific Transformer models (Tu et al., 2021; Zhu et al., 2023; Chen et al., 2026). Transformer-style architectures remain conceptually attractive because they can model long-range dependencies in complex maneuver sequences, but their direct transfer to acupuncture requires task-specific datasets, external validation, and explainability analysis. In adjacent medical AI fields, multimodal frameworks such as HAIM and foundation models such as MUSK illustrate how clinical records, imaging, omics, and sensor-like time series can be fused for prediction and decision support (Soenksen et al., 2022; Xiang et al., 2025).

### 6.3 Machine learning for long-term efficacy prognostication

A key translational value of multimodal AI lies in its potential to bridge the temporal gap between immediate physiological sensory metrics at the initial consultation and long-term clinical endpoints measured weeks or months later.

A retrospective machine learning study quantified Deqi sensation in patients with functional dyspepsia (FD) during their initial acupuncture intervention. By integrating the Massachusetts General Hospital Acupuncture Sensation Scale (MASS) and the Acupuncture Sensation Questionnaire (ASQ), the researchers extracted quantitative features, such as intensity of soreness/distension, grades of sharp pain, and Deqi duration, as model inputs. In this specific study, the authors used a 10-fold cross-validated SVM model to predict whether patients would become higher responders after a 4-week acupuncture regimen; the reported accuracy was 0.84 (84.4% in the full text), with deqi presence, deqi duration, distension, and pain selected as important predictors (Chen et al., 2022b). Because validation strategies differ across studies, the evidence is best interpreted study by study: SVM models based on routine clinical features reported an internal-test accuracy of 0.773, whereas models based on pretreatment functional brain network features reported an accuracy of 0.76 ± 0.03 for responder classification (Yin et al., 2022; 2023).

Related work in chronic pain and autoimmunity further suggests that baseline sensory and biological features can help stratify prognosis, but the evidence should be interpreted cautiously. In an observational pelvic pain cohort, multimodal hypersensitivity derived from Quantitative Sensory Testing (QST) predicted pain outcomes over 4 years and outperformed questionnaire-based generalized sensory sensitivity (Kmiecik et al., 2023). Separately, a network-topology and machine-learning analysis of rheumatoid arthritis used algorithms including RF, GLM, and XGBoost to explore potential immune-related mechanisms of acupuncture treatment (Li F. et al., 2025). Together, these studies support the view that immediate sensory or biological measurements may contribute to responder stratification, but they do not yet establish Deqi as a single validated digital biomarker across diseases.

Despite these reported predictive results, the integration of AI into acupuncture research must address interpretability, validation, and standardization. Deep learning and graph-based models often prioritize predictive performance over clinical explainability, which can limit clinician trust, patient acceptance, and regulatory evaluation (Rajkomar et al., 2019; Liu et al., 2020). Moreover, reviews of AI and deep learning in acupuncture have noted that many studies still rely on self-built, small, single-center datasets, with heterogeneous data formats and limited external validation (Chan et al., 2026; Chen et al., 2026). Future algorithmic development should emphasize explainable AI, external validation, prospective reporting standards, and clinically meaningful error analysis, so that models clarify causal or mechanistic hypotheses rather than merely mapping statistical correlations.

Because the reviewed AI methods differ substantially in input modality and validation maturity, their roles should be compared at the task level rather than treated as interchangeable model choices.

## 7 Future perspectives: engineering challenges, digital twins, and clinical translation pathways

Transitioning from laboratory prototypes to routine, high-throughput acupuncture clinics will require modern intelligent acupuncture systems to progress beyond proof-of-concept demonstrations. Clinical translation therefore depends on resolving multiple interdependent barriers, including patient and operator variability, sensor calibration, long-term stability, sterilization compatibility, affordability, scalable manufacturing, and clinically governed closed-loop control.

### 7.1 Roll-to-roll manufacturing and passive wireless systems

Future omnidirectional monitoring systems for the clinical acupuncture microenvironment must satisfy the rigorous attributes of modern medical consumables, namely, large-area conformal coverage, low manufacturing cost and disposable usage to ensure sterility and prevent cross-infection. Consequently, flexible electronics research is introducing scalable fabrication strategies, including printed electronics, additive manufacturing and roll-to-roll-compatible processes, for the large-scale production of conformal sensing patches, microfluidic channels and polymer nanomeshes (Wei et al., 2024; Yin et al., 2024). By integrating these platforms with flexible printed circuit boards, miniaturized wireless modules and energy-harvesting interfaces, engineers may construct body-area sensing networks that reduce cable drag during clinical needling, although acupuncture-specific cost and reliability data remain limited (Yu et al., 2019; Mahato et al., 2024).

### 7.2 Interface stability and microenvironmental interference

The physiological complexity inherent in clinical populations significantly surpasses the idealized, static conditions typical of materials science laboratories. Primary considerations involve the reconciliation of long-term biocompatibility with the preservation of skin microbiota. While modern flexible modalities have attenuated deep subcutaneous FBR through the reduction of Young’s modulus, protracted epidermal attachment ranging from hours to days introduces substantial clinical risks. These complications include mechanical occlusion of sweat gland ducts, localized hyperthermia of the stratum corneum, and dysbiosis within symbiotic microbial communities. Furthermore, the deployment of subcutaneous probes containing potentially mobile nanomaterials, such as incompletely cross-linked silver nanowires or free carbon nanotubes, necessitates rigorous evaluation of molecular-level nanotoxicity to ensure patient safety.

Equally significant is the degradation of precision electrical characteristics mediated by moisture and high-salinity perspiration. During episodes of sympathetic activation or elevated ambient temperature, intensive perspiration can induce large fluctuations in the dielectric constants of capacitive and piezoresistive sensing layers. Such shifts may precipitate localized micro-short-circuiting within conductive percolation networks, resulting in substantial baseline drift. Mitigating these effects requires breathable porous substrates, stable encapsulation and surface chemistries that resist sweat infiltration while preserving mechanical sensitivity. Superhydrophobic TiO2-ODI modification provides one representative anti-interference strategy, but long-term calibration under clinical perspiration and repeated disinfection remains necessary (Ma et al., 2023; Ates et al., 2022; Mahato et al., 2024).

### 7.3 Closed-loop intelligent robotics and digital twins

With the maturation of front-end multimodal high-frequency sensing networks, high-speed wireless transmission protocols and back-end edge computing decision algorithms, this interdisciplinary field is moving from passive monitoring and validation devices toward intelligent collaborative acupuncture robotic systems capable of environmental awareness and adaptive execution. Current robot-acupuncture reviews and robotic acquisition studies support the feasibility of automated localization, motion tracking and force monitoring, but fully autonomous clinical needling still requires stronger safety validation (Guo et al., 2026; Gao et al., 2025; Li Y.-C. et al., 2025).

The latest generation of minimally invasive, multi-degree-of-freedom acupuncture robotic platforms has achieved high-fidelity replication of expert-level compound maneuvers, such as lifting-thrusting and twirling, in simulator-based validation. A robot arm-assisted acupuncture system with motion and force monitoring reported quantitative validation using trajectory and force metrics, supporting objective assessment of manipulation fidelity (Li Y.-C. et al., 2025). Beyond this electromechanical architecture, the concept of an acupuncture digital twin remains a forward-looking framework that will require prospective validation before clinical deployment.

**FIGURE 3:**
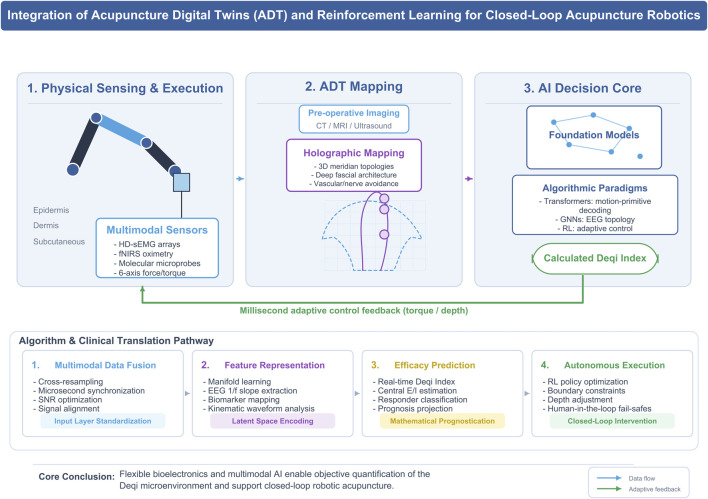
System architecture of the closed-loop precision intelligent acupuncture robotic system empowered by ADT and multimodal AI. The upper framework integrates physical sensing and execution with a robotic arm and multimodal sensors, Acupuncture Digital Twin (ADT) mapping based on pre-operative imaging and patient-specific anatomical reconstruction, and an AI decision core using foundation models and algorithmic paradigms to calculate a Deqi Index. The lower algorithm and clinical translation pathway comprises multimodal data fusion, feature representation, efficacy prediction, and human-in-the-loop autonomous execution. Arrows indicate data flow and millisecond-scale adaptive feedback for torque and depth adjustment.

As shown in Figure 3, the comprehensive framework illustrates the millisecond-scale interaction between physical intervention and patient-specific computational modeling. Top panel: closed-loop control architecture. Physical sensing and execution involve a 6-DOF collaborative robotic arm that performs automated needle manipulation while flexible multimodal bioelectronics acquire real-time kinetic and physiological responses across targeted anatomical layers. ADT mapping then constructs a patient-specific digital model from high-precision pre-operative imaging, such as CT, MRI, or ultrasound, to delineate 3D meridian topologies and deep fascial architectures. The AI processor uses foundation models, including Transformers for motion-primitive decoding and GNNs for EEG topology, to process heterogeneous sensory streams and compute a quantitative Deqi Index. Reinforcement learning algorithms subsequently trigger adaptive safety feedback by adjusting insertion depth and manipulation torque at millisecond timescales. Bottom panel: algorithm and clinical translation pathway. This workflow proceeds from multimodal data fusion and latent-space feature representation to prognostic modeling and human-in-the-loop clinical execution, thereby positioning acupuncture as a more predictable and computable digital medical intervention.

Under this proposed clinical framework, the clinical workflow may be modified rather than fully replaced. The robotic system first uses high-precision pre-operative imaging data, such as CT, MRI or high-frequency surface ultrasound, to construct a patient-specific digital model within local or cloud computing infrastructure. This model may incorporate 3D acupoint-region anatomy, deep nerve trunks, vascular structures surrounding acupoints and fascial-layer geometry. Because anatomical data, image quality and clinical indications vary across patients, such models should be treated as decision-support tools rather than autonomous substitutes for clinician judgment.

During each millisecond-scale control cycle, the digital twin core can process multimodal sensory streams, including needle-tip ultrasonic signals, tissue oxygenation measurements, superficial HD-sEMG arrays and central EEG recordings. AI algorithms may aggregate these data streams to estimate a real-time Deqi Index. The clinical value of this index will depend on calibration, validation and transparent interpretation across different patients and operators.

The integration of reinforcement-learning safety algorithms within the digital twin remains a proposed control strategy. If mechanical energy at the current depth appears insufficient, or if ultrasound and stress feedback predict a risk of approaching arteries or neural bundles, the system could intervene at millisecond timescales. Such intervention may support rapid adjustment of insertion depth, lifting-thrusting amplitude and twirling velocity, but it should remain subject to predefined safety limits and human oversight.

This closed-loop intelligent-control architecture integrates pre-operative anatomical guidance, intra-operative biochemical and mechanical monitoring, and reinforcement-learning-based adaptive feedback. It represents a potential transition for acupuncture from qualitative experiential reasoning toward patient-specific computational modeling supported by edge computing, target-circuit localization and continuous flexible sensing *in vivo*.

### 7.4 Major barriers to clinical translation and regulatory implementation

Despite rapid advances in flexible sensing, multimodal data fusion and intelligent acupuncture systems, clinical translation requires more than demonstrating high sensitivity under controlled laboratory conditions. A clinically viable platform must maintain reproducible performance across heterogeneous patients, practitioners, devices, treatment sessions and healthcare environments. Major barriers include patient variability, inter-operator variability, sensor calibration, long-term stability, sterilization compatibility and implementation cost. These barriers are also emphasized in recent reviews of wearable bioelectronics, multimodal wearable sensors and next-generation wearable, ingestible and implantable biosensing systems (Ates et al., 2022; Mahato et al., 2024; Lu et al., 2023; Mishra et al., 2026; Suryaprabha et al., 2026).

Patient-specific biological variability represents a major source of measurement uncertainty. Age, body mass index, skin thickness, tissue hydration, pigmentation, perspiration, fascial architecture, autonomic state, medication use and disease severity may influence skin-electrode impedance, optical path length, tissue stiffness, baseline biomarker concentrations and subjective Deqi thresholds. Digital-biomarker reviews also emphasize that passive sensor signals can be shaped by behavioral, psychosocial and contextual factors (Shah et al., 2026). Consequently, a signal threshold derived from one population may not be directly transferable to another. Future clinical studies should incorporate individualized baseline measurements, repeated within-subject recordings, anatomical imaging guidance and stratified analyses rather than applying a single universal Deqi threshold. Multicenter validation across diverse demographic and disease populations will be required before a sensor-derived Deqi Index can be considered clinically generalizable.

Inter-operator variability constitutes an equally important challenge because acupuncture remains highly dependent on practitioner-specific technique. Differences in insertion depth, needle angle, lifting-thrusting amplitude, rotational velocity, applied torque, retention time and tactile adjustment can produce distinct mechanical and physiological responses even when the same acupoint and nominal protocol are used. Recent work on acupuncture manipulation measurement and robotic acquisition highlights the need for standardized procedure recording and quantitative manipulation parameters (Gao et al., 2025; Liu X. et al., 2024; Wu et al., 2026). Clinical translation will therefore require standardized procedural reporting, competency-based operator training and explicit recording of practitioner identity and experience. Hierarchical analytical models should distinguish patient-related effects from operator-related effects, while robotic or instrumented reference procedures may provide reproducible benchmarks without eliminating necessary human supervision.

Reliable calibration is essential for comparing measurements across devices and institutions. Flexible piezoresistive, capacitive, optical, electrophysiological and electrochemical sensors exhibit different forms of baseline drift, hysteresis, cross-sensitivity and batch-to-batch variation. Temperature, perspiration, skin deformation, electrode displacement and repeated loading may further alter sensor output during treatment. Reviews of end-to-end wearable sensor design and hybrid multimodal systems emphasize that calibration, temporal synchronization, data quality control and system-level validation are central to reliable deployment (Ates et al., 2022; Mahato et al., 2024; Suryaprabha et al., 2026). Each platform should therefore undergo pre-use zeroing, reference-load testing, temperature and humidity compensation, channel synchronization and periodic recalibration against traceable standards. For multimodal systems, calibration must also include temporal alignment among mechanical, electrophysiological, hemodynamic and biochemical streams.

Long-term stability remains challenging because many flexible devices are optimized for short laboratory demonstrations rather than repeated clinical use. Polymer creep, conductive-network rearrangement, adhesive fatigue, MXene oxidation, liquid-metal leakage, encapsulation failure, electrochemical biofouling and enzyme degradation may progressively reduce sensitivity and increase baseline drift. Recent reviews of wearable, ingestible and implantable biosensors identify biocompatibility, encapsulation, drift control and long-term signal retention as key determinants of translational feasibility (Lu et al., 2023; Mishra et al., 2026; Suryaprabha et al., 2026). Stability should therefore be evaluated not only by mechanical cycling under dry laboratory conditions but also under clinically relevant exposure to sweat, body temperature, repeated bending, skin oils, disinfectants and prolonged attachment. Future studies should report signal retention, calibration drift, mechanical integrity and biocompatibility over the intended clinical lifetime of the device.

Sterilization presents an additional challenge, particularly for needle-integrated biochemical sensors and devices that directly contact compromised skin. Conventional sterilization or disinfection procedures may alter polymer substrates, nanomesh structures, adhesives, surface functional groups, biological recognition layers and electrochemical calibration. A review of polymeric implant sterilization highlights that sterilization method, material chemistry and device architecture must be considered together because heat, radiation and chemical sterilants can affect polymer structure and function (Herczeg and Song, 2022). A practical acupuncture-sensing architecture may therefore separate the system into a sterile disposable patient-contact interface and a reusable wireless processing module. However, post-sterilization sensitivity, selectivity, mechanical integrity, packaging stability and shelf life must be validated before clinical deployment. Sterility assurance and biological evaluation should be considered during material selection and device design rather than addressed only after prototype fabrication.

Affordability will determine whether these technologies can move beyond specialized research centers. The total implementation cost includes not only flexible sensors but also microfabrication, functional nanomaterials, wireless electronics, calibration equipment, data-storage infrastructure, software maintenance, disposable components, clinician training and regulatory testing. Reviews of body-conformable electronics and additive manufacturing for wearable sensors suggest that scalable fabrication, roll-to-roll processing, printed electronics and modular device architectures may reduce production barriers, but cost advantages must be demonstrated in the intended clinical context (Yin et al., 2024; Wei et al., 2024; Mishra et al., 2026). Systems combining HD-sEMG, fNIRS, biochemical probes, imaging and AI may offer rich physiological information but could be impractical for routine acupuncture clinics. Translation should therefore prioritize modular platforms that use only the sensing modalities necessary for a defined clinical decision, together with formal health-economic evaluation.

These practical limitations coexist with broader anatomical, ethical and regulatory concerns. Mechanistic pathways identified in animal models, including the PROKR2-dependent vagal-adrenal circuit, cannot be directly extrapolated to human digital twins without accounting for interspecies differences in fascial architecture and peripheral innervation. Moreover, autonomous adjustment of needle depth and force by reinforcement-learning systems raises unresolved questions regarding safety boundaries, liability, cybersecurity and emergency intervention. Reporting and trial-design guidance for AI interventions, including CONSORT-AI and SPIRIT-AI, emphasizes transparent description of input data, human-AI interaction, error analysis and intended clinical use (Liu et al., 2020; Rivera et al., 2020). Regulatory discussions of AI-enabled digital medical products similarly stress risk management, governance and post-market monitoring (Aboy et al., 2024). Clinical implementation should therefore proceed through multicenter prospective validation, standardized manufacturing and calibration, transparent risk assessment and human-in-the-loop control rather than immediate pursuit of fully autonomous acupuncture.

## 8 Conclusion

This review describes the ongoing shift of acupuncture from an empirical and qualitative practice toward a more measurable digital medical intervention, enabled by flexible multimodal bioelectronics. By replacing legacy rigid sensors with conformable nanomaterials, these interfaces may support high-fidelity, *in situ* decoding of the Deqi microenvironment across biomechanical, hemodynamic, biochemical and electrophysiological dimensions. We further connect localized mechanical inputs to systemic therapeutic outcomes through defined mechanotransduction pathways, notably the Ptgs2+ telocyte-mediated force-immune axis and PROKR2-dependent neural circuits. The integration of AI paradigms, including Transformers and Graph Neural Networks, may help synthesize heterogeneous data into a computable Deqi framework. Looking forward, progress in industrial manufacturing, interface stability, regulatory validation and clinically governed closed-loop control will be necessary for intelligent acupuncture systems to move from exploratory prototypes toward safe translational applications.
